# A Systematic Review of the Effect of Stigmatization on Psychiatric Illness Outcomes

**DOI:** 10.7759/cureus.62642

**Published:** 2024-06-18

**Authors:** Obinna V Chukwuma, Esther I Ezeani, Evelyn O Fatoye, Janet Benjamin, Okelue E Okobi, Chuka G Nwume, Esther N Egberuare

**Affiliations:** 1 Public Health, Purdue University Global, West Lafayette, USA; 2 Family Medicine, Indiana Regional Medical Center (IRMC), Indiana, USA; 3 Primary Care, Lifebridge Health, Baltimore, USA; 4 Medicine, Sumy State University, Sumy, UKR; 5 Internal Medicine, Ross University School of Medicine, Miramar, USA; 6 Family Medicine, Larkin Community Hospital Palm Springs Campus, Miami, USA; 7 Family Medicine, Medficient Health Systems, Laurel, USA; 8 Family Medicine, Lakeside Medical Center, Belle Glade, USA; 9 Family Medicine, University of Port Harcourt, Port Harcourt, NGA; 10 Rehabilitation, Carewest, Calgary, CAN

**Keywords:** discrimination, prejudice, stereotyping, social stigma, psychiatry, mental disorders, negative attributes, stereotypes, stigmatization, stigma

## Abstract

A significant proportion of individuals with psychiatric disorders face dual challenges such as managing the symptoms and disabilities of their conditions and enduring stigma arising from misconceptions about mental illness. This stigma denies them quality-of-life opportunities, such as access to satisfactory healthcare services, better employment, safer housing, and social affiliations. This systematic review aims to evaluate the effect of stigmatization on psychiatric illness outcomes, particularly its influence on treatment adherence, treatment-seeking behavior, and care outcomes. We conducted a systematic review of 39 studies published between 2010 and 2024, focusing on the effects of stigmatization on psychiatric illness outcomes. The review utilized robust methodology following Cochrane guidance and Preferred Reporting Items for Systematic Reviews and Meta-Analyses guidelines, including studies from 2010 to 2024 obtained from databases such as PubMed, Embase, Google Scholar, Web of Science, and SCOPUS. The quality of the included studies was assessed using the Appraisal Tool for Cross-Sectional Studies, with most studies rated as moderate to high quality. The findings indicate that stigma in psychiatric illness is closely associated with several factors, including illness duration (mean effect size = 0.42, p < 0.05), frequency of clinic visits (mean reduction = 2.3 visits/year), and diagnosis of psychotic disorders (OR = 1.78, 95% CI: 1.20-2.65). Stigma manifests through misinformation, prejudice, and discrimination, leading to significant barriers to accessing and adhering to psychiatric treatment, thereby worsening health outcomes. It leads to delays in accessing healthcare, poor adherence to medication and follow-up, and negative psychiatric health outcomes, including disempowerment, reduced self-efficacy, increased psychiatric symptoms, and decreased quality of life. Also, stigma extends to caregivers and healthcare professionals, complicating care delivery. This review highlights the need for effective interventions and strategies to address stigma, emphasizing the importance of educational interventions to mitigate the adverse effects of public stigma. Understanding the multifaceted nature of stigma is crucial for developing targeted approaches to improve psychiatric care outcomes and ensure better mental health services for individuals with mental illnesses.

## Introduction and background

Erving Goffman’s work, “Stigma: Notes on the Management of Spoiled Identity,” published in 1963, inspired researchers to study the causes and outcomes of stigma more extensively [1]. Stigma can be defined as the simultaneous occurrence of status loss, labeling, trivialization, and discrimination or segregation in a given situation [2]. Despite significant advancements in mental health care over the past five decades, efforts to reduce psychiatric stigma have been insufficient [3]. The stigma associated with psychiatric disorders, including within healthcare systems and among healthcare providers, remains a significant barrier to better treatment outcomes and recovery, resulting in poor quality of care for psychiatric patients [4,5]. Stigma negatively affects the willingness of patients and healthcare providers to seek and provide treatment, respectively, leading to poorer health outcomes and increased healthcare costs [5,6]. Severe psychiatric disorders often lead to significant behavioral changes, posing risks to patients, their families, and healthcare providers [7]. These emergencies, though secondary to the psychiatric illness, can lead to fear, prejudice, anger, and exclusion. Common psychiatric emergencies include severe depressive and manic episodes, impaired judgment, substance intoxication, aggressive agitation, withdrawal crises, and severe self-neglect [7]. Effective management of these situations can help reduce or prevent the perpetuation of stigma.

Psychiatric illnesses are among the most widely stigmatized disorders globally, despite evidence that they result from neurochemical imbalances rather than moral failings or witchcraft, as often perceived by the public [8]. Stigmatizing attitudes and labeling of psychiatric illnesses are widespread [9]. The complex nature of mental health stigma is attributed to public misunderstanding, misperception, and lack of information about mental illnesses [10]. Stigma in psychiatric illness involves five key issues: negative attitude, labeling, status loss, discrimination, and segregation [11]. Consequently, the public often views individuals with mental illness as incompetent, dangerous, irresponsible, less human, and unpredictable [10,12].

The effects of stigmatization on individuals with mental illness can be severe, leading to treatment avoidance, incomplete treatment, job insecurity, low self-esteem, and rejection in marriage by families [13-15]. The prevalence of stigma has been noted to vary from one country to another, with the estimated prevalence rates in Canada, Nigeria, and Ethiopia being 24.4%, 22.5%, and 83.5%, respectively [10-15]. Moreover, factors that include poverty, educational status (inability to write and read), poor social support, psychiatric disorder duration of less than one year, lack of employment, substance abuse and use, as well as type of psychiatric illness have been acknowledged to worsen stigmatization among mentally ill persons [7,9,10-14]. Still, stigma creates barriers to recovery and positive treatment outcomes. Recent studies have highlighted perceived stigma as a critical factor undermining patients’ self-esteem, prompting self-isolation and social withdrawal, and deterring individuals from seeking mental health treatment [13,14,16,17]. Stigma also extends to caregivers, including family members and healthcare providers, known as associative stigma [10,17,18]. Associative stigma refers to negative attitudes toward individuals not because of their characteristics but due to their association with someone with a mental illness [19]. Initially applied to family members, this concept now includes mental health professionals due to growing evidence of stigma toward them [10,20,21]. Factors contributing to associative stigma among healthcare professionals include overestimation of coercion in mental health services, public ignorance about mental illness, indistinct boundaries between normality and abnormality in psychiatry, and oversimplification of psychiatric issues [22].

Globally, mental health services are often neglected and under-resourced, especially in developing nations [23]. In developed countries, high levels of stigmatization persist, with nearly 90% of individuals with mental illness reporting experiences of stigma [24,25]. In some Asian countries, cultural values, fear of shame, and strong family values restrict the pursuit of psychiatric treatment [26]. Current research indicates that no country is free from the stigmatization of psychiatric illness [27]. Additionally, projections suggest an increase in psychiatric disorders, including depression, and associated costs worldwide by 2030, highlighting the need for more effective interventions to reverse these trends [28,29]. We conducted a systematic review of existing literature, specifically highlighting studies that explored the consequences of stigmatization on psychiatric illness outcomes.

## Review

Materials and methods

To collect relevant research and peer-reviewed studies published in English, we conducted an in-depth search of various online medical databases, including PubMed, Embase, Google Scholar, Web of Science, and SCOPUS, covering 2010 to 2024. The selected articles included epidemiological studies, health assessment studies with de-identified data, multicenter studies, and published review articles. We identified duplicate data sources by comparing different articles and studies from similar population years. The literature search employed keywords such as stigma, stigmatization, stereotypes, negative attributes, mental disorders, psychiatry, social stigma, stereotyping, prejudice, and discrimination. This search yielded a total of 988 articles.

Inclusion and exclusion criteria

After removing duplicates, we selected relevant articles through a three-phase process. In the first phase, we screened the titles and abstracts. The second phase involved excluding irrelevant articles. In the third phase, we conducted a thorough full-text examination of the selected articles to confirm relevance. Three independent reviewers conducted the article screening process, and any discrepancies were resolved through consultation and consensus.

The inclusion criteria required original studies, such as crossover design studies, randomized controlled trials, and prospective cohort studies, that met the following criteria: scientific publication of original research findings, published in reputable peer-reviewed journals from 2010 to 2024, and written in English. The studies must have investigated stigmatization in psychiatric illness and included individuals with mental illnesses, focusing on the effects of stigma on psychiatric treatment and health outcomes.

We excluded sponsored clinical trials, editorials, and narrative reviews. We also excluded studies that did not use standard tools to assess stigmatization in mentally ill persons or those that assessed stigmatization without relevance to the target populations. Articles that were inaccessible or had unsound materials and methods were also excluded. Following these criteria, 965 articles were excluded.

For this systematic review, we extracted pertinent data from the qualified articles. We collected general study attributes, including the authors’ names, the study year, the year of publication, and the sampling methodologies used. Additionally, we gathered study population attributes such as race, sample size, participants’ gender and age, and follow-up information. We also documented the type of intervention employed, the intervention duration, and the measures used in weight assessment. Finally, we noted the main findings of each study.

We conducted the study selection process using the Preferred Reporting Items for Systematic Reviews and Meta-Analyses (PRISMA) guidelines. The initial database search retrieved 988 article records. After screening, 578 duplicates were removed, and 207 articles were found ineligible through automation. Another 130 records were excluded for various reasons, including misalignment with our study objectives and animal-based studies. Dissertations and studies published in non-peer-reviewed journals were also excluded. We excluded studies initially published in languages other than English, opinion pieces, articles by non-academics, scoping reviews, secondary studies, and non-primary research types.

**Figure 1:**
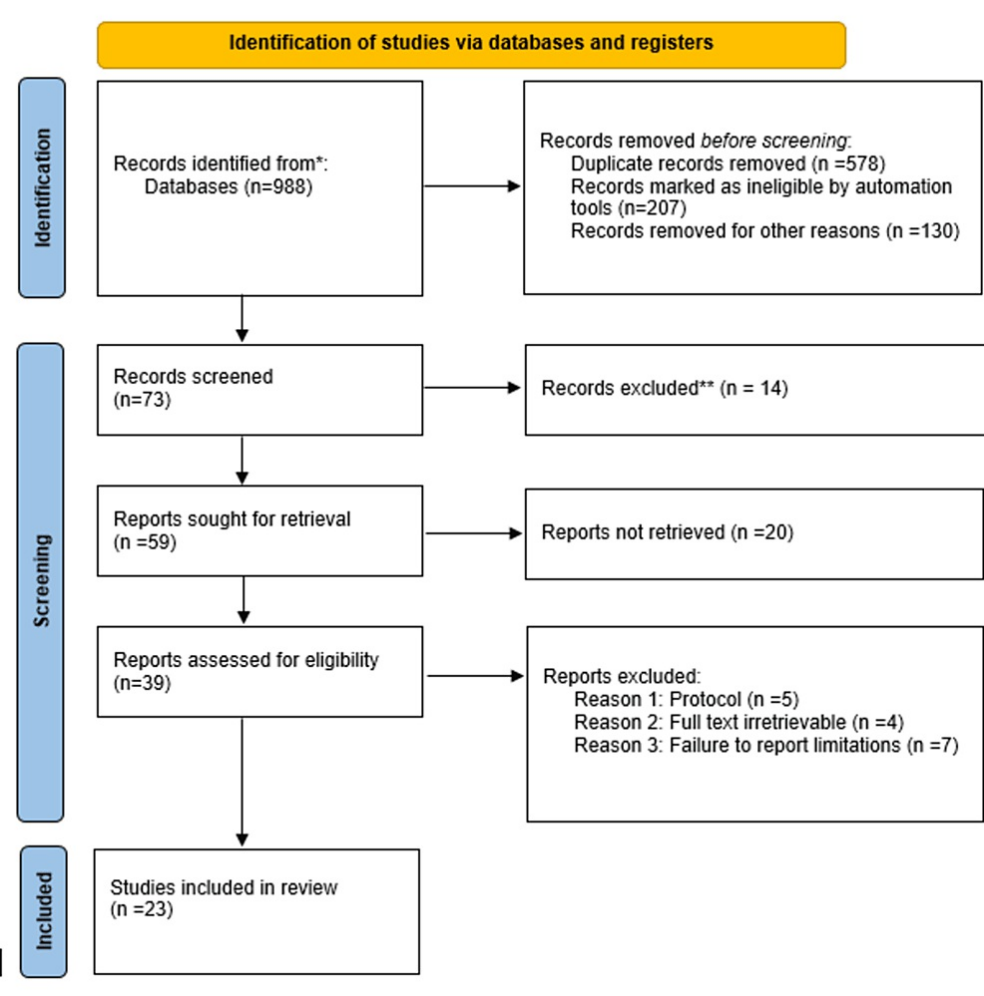
PRISMA flow diagram indicating the study selection process for the studies included in this systematic review. PRISMA: Preferred Reporting Items for Systematic Reviews and Meta-Analyses

Ultimately, 73 eligible articles underwent screening, and 14 were excluded. The remaining 59 articles were sought for retrieval, but 20 were not retrieved. Thus, 39 articles underwent evaluation for eligibility, leading to the exclusion of 16 articles after full-text screening for reasons such as irretrievable full text (four articles), protocol issues (five articles), and failure to report limitations (seven articles). The article selection process is presented in the PRISMA flow diagram in Figure 1.

Quality assessment

We assessed the quality of the included studies in this systematic review using the Appraisal Tool for Cross-Sectional Studies, a 20-item critical appraisal tool designed for cross-sectional studies [30]. Three authors independently evaluated each study, and two additional authors confirmed the assessments. We resolved any potential disagreements through group discussions. Each study received a score of 1 (yes), 0 (no), or “don’t know” for items that were not applicable. Overall, the quality of the included studies ranged from moderate to high, with three studies rated as moderate quality and the remaining 10 as high quality.

Data extraction

We developed a data extraction form to extract pertinent data from the included studies. We collected data on the reviewed studies’ attributes, including the authors’ names, publication year, location, sample size, findings, stigma prevalence, research design, correlates, stigmatization areas, and impacts on the health outcomes of individuals with mental illness. Three authors independently extracted these data, and we resolved any discrepancies through group discussions.

Discussion

The World Health Organization defines stigma as the mark of disgrace, shame, and disapproval resulting from discrimination, rejection, and exclusion of a person from various areas of society [31]. As a social phenomenon, stigma devalues individuals and groups based on specific attributes and characteristics. It comprises three key elements: challenges in knowledge (misinformation or ignorance), behavior (discrimination), and attitudes (prejudice) [32]. These components are interconnected; a lack of knowledge can lead to prejudice and negative attitudes, ultimately resulting in discriminatory behaviors toward mentally ill individuals [33].

Despite advances in educating healthcare professionals and the general public about the neurobiological and genetic underpinnings of psychiatric disorders and substance abuse, stigma remains prevalent. Psychiatric and substance abuse disorders continue to be among the most stigmatized health conditions globally. Interventions aimed at reducing stigma are crucial to removing barriers to care. Approximately 35% of United States residents with severe mental illness and approximately 90% of individuals with substance abuse disorders neither seek nor receive psychiatric treatment [34]. Stigma significantly restricts the use of psychiatric treatment services. For instance, individuals with alcohol use disorder (AUD) who perceive high levels of public stigma are nearly half as likely to seek help compared to those perceiving lower levels of stigma [35,36].

Erving Goffman first described stigmatization in psychiatric health in 1963, identifying it as any attribute or characteristic that taints, devalues, discredits, and brings shame to a mentally ill individual [1,32]. Goffman’s work has profoundly influenced subsequent studies in this field, leading to explorations of stigmatization across various contexts and cultural backgrounds. Stigmatization in psychiatric health is strongly influenced by contextual and cultural value systems, which vary across contexts and over time. However, many researchers agree with Goffman’s basic description of stigma in psychiatric health, which includes stereotyping, prejudice, social isolation, labeling, ignorance, rejection, discrimination, low self-esteem, marginalization, status loss, and low self-efficacy [37-39]. Therefore, stigmatization in psychiatric health is defined as the social disapproval, disgrace, and social discrediting of mentally ill individuals [40,41].

The literature identifies several dimensions and types of stigma in psychiatric illness, including self-stigmatization, professional stigmatization, institutional stigmatization, and public stigmatization. These types of stigma occur in different contexts and settings, each having different impacts on psychiatric health outcomes. Self-stigmatization, or internalized stigma, refers to an individual’s negative attitude toward their psychiatric disorder [37,42]. The findings of this systematic review indicate that self-stigmatization directly correlates with negative psychiatric health outcomes, including disempowerment, failure to seek treatment, reduced self-efficacy, and decreased quality of life, all of which adversely affect treatment and health outcomes [43].

Public stigma involves the general public’s negative attitudes toward mentally ill individuals, often based on fear, prejudice, misunderstanding, and misconception [37,42]. Associated with public stigma is perceived stigma, which refers to mentally ill individuals’ beliefs about others’ attitudes toward mental illness. Recent studies have shown that public stigma significantly impacts mental health and treatment outcomes, including discrimination within public agencies and workplaces [44].

Professional stigmatization occurs when healthcare professionals exhibit stigmatizing attitudes toward mentally ill patients. These attitudes stem from fear and misunderstanding of psychiatric disorders’ causes and symptoms, as well as experiences of stigmatization from the general public and other healthcare professionals due to their association with mentally ill individuals [37]. Professional stigmatization is particularly concerning as it can affect the treatment and care provided to mentally ill patients, including treatment for physical illnesses, thereby impacting patients’ well-being and recovery [37].

Institutional stigmatization refers to negative attitudes embedded in an organization’s culture and policies toward stigmatized individuals, including those who are mentally ill [37,42,45-48]. Institutional stigma can be reinforced by legal frameworks, professional practices, and public policies, becoming deeply entrenched in society [44].

Nevertheless, stigma often reinforces itself through various dimensions, emerging at intra- and interpersonal levels and enacted through regulations, policies, and laws [49]. It extends beyond the stigmatized individuals, echoing through the wider community and affecting procedures and policies guiding treatment, including the behavior of healthcare professionals [50]. Pescosolido et al. devised a Framework for Integrating Normative Influences on Stigma to address these divergent forms of stigma, which posits that different levels of social life create normative expectations that lead to stigmatization [38]. The framework identifies sources of psychiatric illness-related stigma at micro (internalized stigma), macro (structural stigma), and meso (public stigma) levels, focusing on how to impact psychiatric disorder treatment.

Within mental healthcare, stigma remains a significant concern for mentally ill individuals and their families. Several studies reveal that stigmatization in psychiatric illness hinders access to appropriate and professional psychological and mental treatment, potentially worsening patients’ conditions and leading to multiple hospital admissions [16,51,52]. Patients often describe the stigma and prejudice they experience as being as detrimental as their psychiatric disorder symptoms, affecting both their public and private lives [53]. Stigma also impacts the families of patients and healthcare providers working in psychiatric units [15,54-56]. Reducing stigma in mental healthcare is crucial for improving psychiatric outcomes and should be a public health priority. Future research should focus on developing and evaluating interventions to mitigate stigma and its effects on mental health.

This study aimed to evaluate the impact of stigmatization on psychiatric illness outcomes. Our findings confirm that stigmatization significantly worsens psychiatric symptoms and reduces treatment-seeking behaviors, aligning with the study’s objectives [57]. For example, Yanos et al. found that self-stigma negatively impacts treatment and recovery in individuals with chronic psychiatric disorders, leading to reduced hopes, low self-esteem, increased psychiatric symptoms, decreased adherence to treatment regimens, and challenges in social relationships and work [55]. Similarly, a study by Oexle et al. involving 222 mentally ill patients over 24 months found that higher levels of self-stigma were associated with poor recovery after one to two years [58]. Da Silva et al. assert that stigmatization in psychiatric illness has pervasive effects, impacting political support, local psychiatric healthcare services, charitable fundraising, and underfunding of crucial studies and clinical trials for mental health compared to other medical conditions [54,57].

Healthcare providers may also stigmatize individuals with psychiatric disorders, as noted by Thornicroft et al. Biases among healthcare professionals can lower the likelihood of mentally ill individuals receiving appropriate treatment or referrals to specialty care services [59]. Interventions aimed at reducing healthcare provider-related stigma show promise. For example, a 2021 study found that testimonies from psychiatric patients who recovered due to treatment reduced stigma among medical students [54]. Such interventions can enhance the probability and quality of psychiatric treatment and care services offered to mentally ill individuals [60].

Self-stigma, the internalization of stigma, significantly decreases the interest of mentally ill individuals in seeking help for their psychiatric disorders [61,62]. Studies indicate that self-stigma negatively impacts recovery by reducing self-worth and self-esteem, diminishing hopes for recovery, impairing social relationships, and worsening psychiatric symptoms [54,63]. Self-stigma also increases avoidant coping and suicide ideation risks while reducing vocational functioning and treatment adherence [15,55]. For instance, a 2019 study revealed that more than 17% of individuals with AUD and other substance use disorders did not seek treatment due to concerns about negative opinions from their communities and neighbors [15,34]. Additionally, painful experiences of prejudice and discrimination within healthcare contexts are major causes of treatment-seeking avoidance among individuals with mental disorders. The stigma surrounding AUD and substance abuse disorders is internalized, causing ongoing shame and distress, leading to further substance abuse [64].

Several studies have disclosed the existence of a negative correlation between stigma and recovery-related outcomes, as well as a positive correlation between stigma, suicidal ideation, and depression [65,66]. Additionally, cognitive impairments, avoidant coping strategies, and dysfunctional attitudes are positively linked to stigma in psychiatric illness. Dubreucq et al. noted that insight into mental illness significantly moderates internalized stigma [67]. Experienced and perceived stigma, as well as perceived cognitive dysfunction, indirectly affect functional and clinical outcomes through self-stigma [57,67].

Baseline self-stigma correlates with poorer recovery outcomes and reduced benefits from vocational rehabilitation at follow-up [66,68]. Reductions in self-stigma have been linked to improvements in suicidality, depression, self-esteem, attitudes toward medication, social functioning, and quality of life at follow-up [67]. Various studies report that stigmatization adversely affects “what matters most” within the local social world by threatening an individual’s ability to meet social expectations, such as participating in life activities like marriage and work [69,70] and impacting the entire family’s socioeconomic status and moral standing. Although self-stigma is reportedly more widespread in non-Western nations than in Western nations, this observation remains unproven.

Several studies that we assessed in this systematic review explored the effects of stigma in psychiatric illness on treatment duration and reported mixed outcomes [71-74]. For example, a study on veterans from the Afghanistan and Iraq wars found that higher degrees of perceived stigma were linked to, but did not predict, the number of outpatient psychiatric visits made by participants who registered for treatment [72]. However, higher perceived stigma predicted the likelihood of completing a significant treatment episode, especially in patients with post-traumatic stress disorder (PTSD) and depression [72]. Another study found that higher stigma scores forecasted a greater likelihood of completing eight or more PTSD-associated psychotherapy visits in 260 veterans who began psychotherapy [73]. Conversely, a study of 103 participants in a residential substance use therapy program found that higher levels of self-stigma resulted in longer hospitalization durations in treatment facilities [74].

A 2022 study indicated that different types of stigma correlate differently with depressive symptoms [75]. In a sample of 255 outpatients with severe psychiatric disorders, higher rates of reported self-stigma at baseline predicted greater reports of depressive symptoms four months later [75]. Higher scores on self-stigma subscales measuring alienation, social withdrawal, and stereotype endorsement significantly predicted severe depressive symptoms, although scores for public stigma did not [72,74,75].

Stigma’s effects have also been evaluated in relation to the quality of life and functioning of mentally ill individuals. Several studies in this review addressed the issue of stigma and its impact on quality of life and functioning, indicating a negative correlation between stigmatization and these outcomes. For example, a study of outpatient and inpatient psychiatric health service users in Germany found that higher levels of stigma experienced by patients predicted poorer mental health outcomes nine months later [76]. Another study disclosed that in individuals with schizoaffective disorders and schizophrenia, baseline stereotype endorsement ratings significantly predicted changes in vocational functioning at five months, as evaluated using the Quality of Life Scale’s Instrumental Functioning subscale [77]. Greater stereotype endorsement was linked to reduced improvements in functioning [55]. A study focusing on mental health service users with major mood disorders and schizophrenia with higher self-stigma rates at baseline found that increased stigma levels were linked to lower quality of life scores [78]. However, since the study sample was selected based on higher reported self-stigma levels, it is unclear if the findings are generalizable to the broader population of mentally ill individuals.

Limitations

This systematic review has several limitations, primarily due to the heterogeneity in the description of stigma in mental illness, as well as variations in contexts, methodologies, and reported findings. This diversity complicates comparable data extraction. Only a limited number of the reviewed articles reported longitudinal outcomes, and few studies were conducted within psychiatric rehabilitation clinics or centers. Additionally, a significant proportion of the included articles (12 articles) focused on self-stigma, suggesting that other forms of stigma might also have wide-ranging effects on psychiatric illness outcomes. However, the emphasis on self-stigma in this review provides a detailed understanding of the effects of stigmatization on individuals with mental illnesses.

## Conclusions

This systematic review provides a thorough examination of the concept of stigma and its implications within the domain of psychiatric illnesses. This knowledge is vital for developing effective interventions and strategies to improve psychiatric care outcomes for individuals with mental illnesses. The literature review presented has revealed that regardless of the progress made in understanding the dimensions of stigma associated with psychiatric illnesses and the processes through which public stereotypes and prejudices translate into discriminatory behaviors, stigma in psychiatric disorders remains widespread globally, significantly affecting mental healthcare outcomes. Mental stigma is particularly prevalent among younger individuals, the unemployed, females, single individuals, those residing in rural areas, and those with lower socioeconomic and educational levels. Stigma in psychiatric illness correlates with the duration of illness, number of clinic visits or hospital admissions, and diagnosis of a psychotic disorder. It also leads to delays in accessing healthcare, poor medication adherence, and follow-up issues, all of which impact the recovery process and duration. Effective interventions to address stigma are critical for improving mental health services and outcomes. Educational interventions are essential to prevent the adverse effects of public stigma on psychiatric illness.
